# Supplementary material for: Heat adaptation of phage T7 under an extended genetic code
Source: Virus Evol. 2021 Dec 1;7(2):veab100. doi: 10.1093/ve/veab100 (PMC8923235; doi:10.1093/ve/veab100)
Supplement: veab100_Supp [file veab100_supp.zip › Sequencing_Analysis.zip!/Sequencing_Analysis/S2/evidence/JC_42.html]

BRESEQ :: Evidence


| New junction evidence | | | | | | | | | | | |
| --- | --- | --- | --- | --- | --- | --- | --- | --- | --- | --- | --- |
|  | | seq id | position | reads (cov) | reads (cov) | score | skew | freq | annotation | gene | product |
| \* | ? | NC\_001604.1 | 295 = | 7072 (1.340) | 31 (0.010) | 13/560 | 48.9 | 0.47% | intergenic (–/–) | *–/–* | –/– |
|  | ? | NC\_001604.1 | = 281 | 6648 (1.350) |  |  |  |  | intergenic (–/–) | *–/–* | –/– |
| Rejected: Coverage evenness skew score above cutoff. | | | | | | | | | | | |
| Rejected: Frequency below/above cutoff threshold. | | | | | | | | | | | |

Only 100 of 843 total aligned reads displayed.  

`TTACTGCATGTTACTTCATGTTGTCAATACCTGTTTTTCGTGCGACTTATCAGGCTGTCTACTTATCCGGTTGACCCCGGTGTCGATTGGGATGGCTATTCGCCGTGTCCCTCTCGATGGCTGTAAGTATCCTATAGGTTAGACTTTAAGTCAATACTCTTTTTGATAAATTTTAAATTAATCTTTTAAGTCTCTTTAAGTTGTCTCTCCTTA‑TAGTGAGTTGTATTAATAAAGACAATGATTAAAGGTTATCACTTTATGGCTAACAACAAGGCGTCTTTAGGTCTGGTCTTTATGTAG‑TGTCTTTA‑‑‑‑‑GGTCTGGTCTTTA‑‑‑‑‑‑‑‑‑‑‑‑‑‑‑‑‑‑‑‑‑‑‑‑‑‑‑‑‑‑‑‑‑‑‑‑‑‑‑‑‑‑‑‑‑‑‑‑‑‑‑‑‑‑‑‑‑‑‑‑‑‑‑‑‑‑‑‑‑‑‑‑‑‑‑‑‑‑‑‑‑‑‑‑‑‑‑‑‑‑‑‑‑‑‑‑‑‑‑‑‑‑‑‑‑‑‑‑‑‑‑‑‑‑‑‑‑‑‑‑‑‑‑‑‑‑‑‑‑‑‑‑‑‑‑‑‑‑‑‑‑‑‑‑‑‑‑‑‑‑‑‑‑‑‑‑‑‑‑‑‑‑‑‑‑‑‑‑‑‑‑‑‑‑‑‑‑‑‑‑‑‑‑‑‑‑‑‑‑‑‑‑‑‑‑‑‑‑‑‑‑‑‑‑‑‑‑‑‑‑‑‑‑‑‑‑‑‑‑‑‑‑‑‑‑‑‑‑‑‑‑‑‑‑‑‑‑‑‑‑‑‑‑‑‑‑‑‑‑‑‑‑‑‑‑‑‑‑‑‑‑‑‑‑‑‑‑‑‑‑‑‑‑‑‑‑‑‑‑‑‑‑‑‑‑‑‑‑‑‑‑‑‑‑‑  <  NC_001604.1/615‑295`  
`‑‑‑‑‑‑‑‑‑‑‑‑‑‑‑‑‑‑‑‑‑‑‑‑‑‑‑‑‑‑‑‑‑‑‑‑‑‑‑‑‑‑‑‑‑‑‑‑‑‑‑‑‑‑‑‑‑‑‑‑‑‑‑‑‑‑‑‑‑‑‑‑‑‑‑‑‑‑‑‑‑‑‑‑‑‑‑‑‑‑‑‑‑‑‑‑‑‑‑‑‑‑‑‑‑‑‑‑‑‑‑‑‑‑‑‑‑‑‑‑‑‑‑‑‑‑‑‑‑‑‑‑‑‑‑‑‑‑‑‑‑‑‑‑‑‑‑‑‑‑‑‑‑‑‑‑‑‑‑‑‑‑‑‑‑‑‑‑‑‑‑‑‑‑‑‑‑‑‑‑‑‑‑‑‑‑‑‑‑‑‑‑‑‑‑‑‑‑‑‑‑‑‑‑‑‑‑‑‑‑‑‑‑‑‑‑‑‑‑‑‑‑‑‑‑‑‑‑‑‑‑‑‑‑‑‑‑‑‑‑‑‑‑‑‑‑‑‑‑‑‑‑‑‑‑‑‑‑‑‑‑‑‑‑‑‑‑‑‑‑‑‑‑‑‑‑‑‑‑‑‑‑‑‑‑‑‑‑‑‑‑‑‑‑‑‑‑‑‑‑‑‑‑gtcttta‑‑‑‑‑ggtctggtctttaTGTTTAAACTTTAAGATAGGCGTTGACTTGATGGGTCTTTAGGTGTAGGCTTTAGGTGTTGGCTTTAGGATGGACGTTAGGAGGTGACTTTAGGAGGATACTTTAGGAGACTGTAACAGATA‑GGGACACAGAGAGACACTCAAGGTAACACCCAAAGACAAACCCAAGGGTCATCCCCAACCCTTAGGGAACCCTCAAGGTCAACCGAAGGTTGACTTTAGGTGATTGGCTGGGCTTTAGGTACCCCCTATGGGGGAACTTTAGGTCCGTACACTGTGAGA‑‑‑‑‑‑‑‑‑‑‑‑‑  <  NC_001604.1/281‑1`  

                                                                                                                                                                                                                                                                                                                                                                                                                                                                                                                                                                                                                                                 
 `TACTGCATGTTACTTCATGTTGTCAATACCTGTTTTTCGTGCGACTTATCAGGCTGTCTACTTATCCGGTTGACCCCGGTGTCGATTGGGATGGCTATTCGCCGTGTCCCTCTCGATGGCTGTAAGTATCCTATAGGTTAGACTTTAAGTCAATACTCTTTTTGATAAATTTTAAATTAATCTTTTAAGTCTCTTTAAGTTGTCTCTCCTTA‑TAGTGAGTTGTATTAATAAAGACAATGATTAAAGGTTATCACTTTATGGCTAACAACAAGGCGTCTTTAGGTCTGGTCTTTATGTAG‑TG                                                                                                                                                                                                                                                                                                                                 <  1:29642/301‑1`  
 `TACTGCATGTTACTTCATGTTGTCAATACCTGTTTTTCGTGCGACTTATCAGGCTGTCTACTTATCCGGTTGACCCCGGTGTCGATAGGGATGGCTATTCGCCGTGTCCCTCTCGATGGCTGTAAGTATCCTATAGGTTAGACTTTAAGTCAATACTCTTTTTGATAAATTTTAAATTAATCTTTTAAGTCTCTTTAAGTTGTCTCTCCTTA‑TAGTGAGTTGTATTAATAAAGACAATGATTAAAGGTTATCACTTTATGGCTAACAACAAGGCGTCTTTAGGTCTGGTCTTTATGTAG‑TG                                                                                                                                                                                                                                                                                                                                 <  1:822321/301‑1`  
  `agacagTGTTACTTCATGTTGTCAATACCTGTTTTTCGTGCGACTTATCAGGCTGTCTACTTATCCGGTTGACCCCGGTGTCGATTGGGATGGCTATTCGCCGTGTCCCTCTCGATGGCTGTAAGTATCCTATAGGTTAGACTTTAAGTCAATACTCTTTTTGATAAATTTTAAATTAATCTTTTAAGTCTCTTTAAGTTGTCTCTCCTTA‑TAGTGAGTTGTATTAATAAAGACAGTGATTAAAGGTTATCACTTTATGGCTAACAACAAGGCGTCTTTAGGTCTGGTCTTTATGTAG‑TGT                                                                                                                                                                                                                                                                                                                                <  1:201106/295‑1`  
  `ACTGCATGTTACTTCATGTTGTCAATACCTGTTTTTCGTGCGACTTATCAGGCTGTCTACTTATCCGGTTGACCCCGGTGTCGATTGGGATGGCTATTCGCCGTGTCCCTCTCGATGGCTGTAAGTATCCTATAGGTTAGACTTTAAGTCAATACTCTTTTTGATAAATTTTAAATTAATCTTTTAAGTCTCTTTAAGTTGTCTCTCCTTA‑TAGTGAGTTGTATTAATAAAGACAATGATTAAAGGTTATCACTTTATGGCTAACAACAAGGCGTCTTTAGGTCTGGTCTTTATGTAG‑TGT                                                                                                                                                                                                                                                                                                                                >  1:510659/1‑301`  
   `CTGCATGTTACTTCATGTTGTCAATACCTGTTTTTCGTGCGACTTATCAGGCTGTCTACTTATCCGGTTGACCCCGGTGTCGATTGGGATGGCTATTCGCCGTGTCCCTCTCGATGGCTGTAAGTATCCTATAGGTTAGACTTTAAGTCAATACTCTTTTTGATAAATTTTAAATTAATCTTTTAAGTTTCTTTAAGTTGTCTCTCCTTA‑TATTGAGTTATATTAATAAAGACAATGATTAAAGGTTATCACTTTATGGCTAACAACAAGGCGTCTTTAGGTCTGGTCTTTATGTAG‑TGTt                                                                                                                                                                                                                                                                                                                               >  1:764125/1‑300`  
   `CTGCATGTTACTTCATGTTGTCAATACCTGTTTTTCGTGCGACTTATCAGGCTGTCTACTTATCCGGTTGACCCCGGTGTCGATTGGGATGGCTATTCGCCGTGTCCCTCTCGATGGCTGTAAGTATCCTATAGGTTAGACTTTAAGTCAATACTCTTTTTGATAAATTTTAAATTAATCTTTTAAGTCTCTTTAAGTTGTCTCTCCTTA‑TAGTGAGTTGTATTAATAAAGACAATGATTAAAGGTTATCACTTTATGGCTAACAACAAGGCGTCTTTAGGTCTGGTCTTTATGTAG‑TGTt                                                                                                                                                                                                                                                                                                                               >  1:642857/1‑300`  
     `GCATGTTACTTCATGTTGTCAATACCTTTTTTTCGTGCGACTTATCAGGCTGTCTACTTATCCGGTTGACCCCGGTGTCGATTGGGATGGCTATTCGCCGTGTCCCTCTCGATGGCTGTAAGTCTCCTATAGGTTAGACTTTAAGTCAATCCTCTTTTTGATAAATTTTAAATTAATCTTTTAAGTCTCTTTAAGTTGTCTCTCCTTA‑TAGTTAGTTTTATTAATAAAGACAATGATTAAAGGTTATCACTTTATGGCTAACAACAAGGCGTCTTTAGGTCTGGTCTTTATGTAGCTGTCT                                                                                                                                                                                                                                                                                                                              >  1:571413/1‑301`  
     `GCATGTTACTTCATGTTGTCAATACCTGTTTTTCGTGCGACTTATCAGGCTGTCTACTTATCCGGTTGTCCCCGGTGTCGATTGGGATGGCTATTCGCCGTGTCCCTCTCGATGGCTGTAAGTATCTTATAGGTTAGACTTTAAGTCAATACTCTTTTTGATAAATTTTAAATTAATCTTTTAAGTCTCTTTATGTTGTCTCTTCTTT‑TAGTGAGTTGTATTAATATAGACAATGATTAAAGGTTATCAACTTATGGCTAACAACAAGGCGTATTTAGGTCTGTTCTTTATGTAG‑TGTCTT                                                                                                                                                                                                                                                                                                                             >  1:286336/1‑301`  
     `GCATGTTACTTCATGTTGTCAATACCTGTTTTTCGTGCGACTTATCAGGCTGTCTACTTATCCGGTTGACCCCGGTGTCGATTGTGATGGCTATTCGCCGTGTCCCTCTCGATGGCTGTAAGTATCCTATAGGTTAGACTTTAAGTCAATACTCTCTTTGATAAATTTTAAATTAATCTTTTAAGTCTCTTTAAGTTGTCTCTCCTTA‑TAGTGAGTTGTATTAATAAAGACAATGATTAAAGGTTATCACTTTATGGCTAACAACAAGGCGTCTTTAGGTCTGGTCTTTATGTAG‑TGTCTT                                                                                                                                                                                                                                                                                                                             >  1:687733/1‑301`  
     `GCATGTTACTTCATGTTGTCAATACCTGTTTTTCGTGCGACTTATCAGGCTGTCTACTTATCCGGTTGACCCCGGTGTCGATTGGGATGGCTATTCGCCGTTTCCCTCTCGATGGCTGTAAGTATCCTATAGGTTAGACTTTAAGTCAATACTCTTTTTGATAAATTTTAAATTAATCTTTTAAGTCTCTTTAAGTTGTCTCTCCTTA‑TAGTGAGTTGTATTAATCATGACAATGATTAAAGGTTATCACTTTTTGGCTAACAACAAGCCGTCTTTAGGTCTGGTCTTTATGTTG‑TGTCTT                                                                                                                                                                                                                                                                                                                             >  1:3147/1‑301`  
     `GCATGTTACTTCATGTTGTCAATACCTGTTTTTCGTGCGACTTATCAGGCTGTCTACTTATCCGGTTGACCCCGGTGTCGATTGGGATGGCTATTCGCCGTGTCCCTCTCGATGGCTGTAAGTATCCTATAGGTTAGACTTTAAGTCAATACTCTTTTTGATAAATTTTAAATTAATCTTTTAAGTCTCTTTCAGTTGTCTCTCCTTA‑TAGTTAGTTGTATTAATAAATACAATGATTAAAGGTTATCACTTTATGGCTAACAACAAGGCGTCTTTAGGTCTGGTCTTTATGTAG‑TGTCTT                                                                                                                                                                                                                                                                                                                             >  1:54987/1‑301`  
     `GCATGTTACTTCATGTTGTCAATACCTGTTTTTCGTGCGACTTATCAGGCTGTCTACTTATCCGGTTGACCCCGGTGTCGATTGGGATGGCTATTCGCCGTGTCCCTCTCGATGGCTGTAAGTATCCTATAGGTTAGACTTTAAGTCAATACTCTTTTTGATAAATTTTAAATTAATCTTTTAAGTCTCTTTAAGTTGTCTCTCCTTA‑TAGTGAGTTGTATTAATAAAGACAATGATTAAAGGTTATCACTTTATGGCTAACAACAAGGCGTCTTTAGGTCTGGTCTTTATGTAG‑TGTCTT                                                                                                                                                                                                                                                                                                                             >  1:403376/1‑301`  
     `GCATGTTACTTCATGTTGTCAATACCTGTTTTTCGTGCGACTTATCAGGCTGTCTACTTATCCGGTTGACCCCGGTGTCGATTGGGATGGCTATTCGCCGTGTCCCTCTCGATGGCTGTAAGTATCCTATAGGTTAGACTTTAAGTCAATACTCTTTTTGATAAATTTTAAATTAATCTTTTAAGTCTCTTTAAGTTGTCTCTCCTTA‑TAGTGAGTTGTATTAATAAAGACAATGATTAAAGGTTATCACTTTATGGCTAACAACAAGGCGTCTTTAGGTCTGGTCTTTATGTAG‑TGTCTT                                                                                                                                                                                                                                                                                                                             >  1:492798/1‑301`  
     `GCATGTTACTTCATGTTGTCAATACCTGTTTTTCGTGCGACTTATCAGGCTGTCTACTTATCCGGTTGAACCCGGTGTCGATTGGGATGGCTATTCGCCGTGTCCCTCTCGATGGCTGTAAGTATCCTATAGGTTAGACTTTAAGTCAATACTATTTTTGATAAATTTTAAATTAATCTTTTAAGTCTCTTTAATTTGTCTCTACTTA‑TAGTTTGTTGTATTATTCAAGAACATGATTACAGGTTATCACTTTACGTCTAACAACAATGCGTCTTTATGTCTGGTCTTTATGTTG‑TTTCTT                                                                                                                                                                                                                                                                                                                             >  1:120788/1‑301`  
      `tATGTTACTTCATGTTGTCAATACCTGTTTTTCGTGCGACTTATCAGGCTGTCTACTTATCCGGTTGACCCCGGTGTCGATTGGGATGGCTATTCGCCGTGTCCCTCTCGATGGCTGTAAGTATCCTATAGGTTAGACTTTAAGTCAATACTCTTTTTGATAAATTTTAAATTAATCTTTTAAGTCTCTTTAAGTTGTCTCTCCTTA‑TAGTGAGTTGTATTAATAAAGACAATGATTAAAGGTTATCACTTTATGGCTAACAACAAGGCGTCTTTAGGTCTGGTCTTTATGTAG‑TGTCTTT                                                                                                                                                                                                                                                                                                                            <  1:109891/300‑1`  
       `ATGTTACTTCATGTTGTCAATACCTGTTTTTCGTGCGACTTATCAGGCTGTCTACTTATCCGGTTGACCCCGGTGTCGATTTGGATGGCTATTCGCCGTGTCCCTCTCGCTGGCTGTAAGTATCCTATAGGTTAGACTTTAAGTCCATACTCTTTTTGATAAATTTTAAATTAATCTTTTAAGTCTCTTTAAGTTGTCTCTCCTTA‑TAGTGAGTTGTATTAATAAAGACAATGATTAAAGGTTATCACTTTATGGCTAACAACAAGGCGTCTTTAGGTCTGGTCTTTATGTAG‑TTTCTgtt                                                                                                                                                                                                                                                                                                                           >  1:545023/1‑298`  
       `ATGTTACTTCATGTTGTCAATACCTGTTTTTCGTGCGACTTATCAGGCTGTCTACTTATCCGGTTGACCCCGGTGTCGATTGGGATGGCTATTCGCCGTGTTCCTCTCGATGGCTGTAAGTATCCTATAGGTTAGACTTTAAGTCAATACTCTTTTTGATAAATTTTAAATTAATCTTTTAAGTCTCTTTAAGTTGTCTCTCCTTA‑TAGTGAGTTGTATTAATAAAGACAATGATTAAAGGTTATCACTTTATGGCTAACAACAAGGCGTCTTTAGGTCTGGTCTTTATGTAG‑TGTCTTTA                                                                                                                                                                                                                                                                                                                           >  1:606593/1‑301`  
       `ATGTTACTTCATGTTGTCAATACCTGTTTTTCGTGCGACTTATCAGGCTGTCTACTTATCCGGTTGACCCCGGTGTCGATTGGGATGGCTATTCGCCGTGTCCCTCTCGATGGCTGTAAGTATCCTATAGGTTAGACTTTAAGTCAATACTCTTTTTGATAAATTTTAAATTAATCTTTTAAGTCTCTTTAAGTTGTCTCTCCTTA‑TAGTGAGTTGTATTAATAAAGACAATGATTAAAGGTTATCACTTTATGGCTAACAACAAGGCGTCTTTAGGTCTGGTCTTTATGTAG‑TGTCTTTg                                                                                                                                                                                                                                                                                                                           >  1:719213/1‑300`  
       `ATGTTACTTCATGTTGTCAATACCTGTTTTTCGTGCGACTTATCAGGCTGTCTACTTATCCGGTTGACCCCGGTGTCGATTGGGATGGCTATTCGCCGTGTCCCTCTCGATGGCTGTAAGTATCCTATAGGTTAGACTTTAAGTCAATACTCTTTTTGATAAATTTTAAATTAATCTTTTAAGTCTCTTTAAGTTGTCTCTCCTTA‑TAGTGAGTTGTATTAATAAAGACAATGATTAAAGGTTATCACTTTATGGCTAACAACAAGGCGTCTTTAGGTCTGGTCTTTATGTAG‑TGTCTTTA                                                                                                                                                                                                                                                                                                                           >  1:668837/1‑301`  
       `ATGTTACTTCATGTTGTCAATACCTGTTTTTCGTGCGACTTATCAGGCTGTCTACTTATCCAGTTGACCCCGGTGTCGATTGGGATGGCTATTCGCCGTGTCCCTCTCGATGGCTGTAAGTATCCTATAGGTTAGACTTTAAGTCAATACTCTTTTTGATAAATTTTAAATTAATCTTTTAAGTCTCTTTAAGTTGTCTCTCCTTA‑TAGTGAGTTGTATTAATAAAGACAATGATTAAAGGTTATCACTTTATGGCTAACAACAAGGCGTCTTTAGGTCTGGTCTTTATGTAG‑TGTCTTTt                                                                                                                                                                                                                                                                                                                           >  1:745616/1‑300`  
        `TGTTACTTCATGTTGTCAATACCTGTTTTTCGTGCGACTTATCAGGCTGTCTACTTATCCGGTTGACCCCGGTGTCGATTGGGATGGCTATTCGCCGTGTCCCTCTCGATGGCTGTAAGTATCCTATAGGTTAGACTTTAAGTCAATACTCTTTTTGATAAATTTTAAATTAATCTTTTAAGTCTCTTTAAGTTGTCTCTCCTTA‑TAGTGAGTTGTATTAATAAAGACAATGATTAAAGGTTATCACTTTATGGCTAACAACAAGGCGTCTTTAGGTCTGGTCTTTATGTAG‑TGTCTTTA‑‑‑‑‑G                                                                                                                                                                                                                                                                                                                     >  1:356439/1‑301`  
         `tTTACTTCATGTTGTCAATTCCTGTTTTTCGTGCGACTTATCAGGCTGTCTACTTATCCGGTTGACCCCGGTGTCGATTGGGATGGCTATTCGCCGTGTCCCTCTCGATGGCTGTAAGTATCCTATAGGTTAGACTTTAAGGCAATACTCTTTTTGATAAATTTTAAATTAATCTTTTAAGTCTCTTTAAGTTGTCTCTCCTTA‑TAGTGAGTTGGATTAATAAAGACAATGATTAAAGGTTATCACTTTATGGCTAACAACAAGGCGTCTTTAGGTCTGGTCTTTATGTAG‑TGTCTTTA‑‑‑‑‑GG                                                                                                                                                                                                                                                                                                                    <  1:101690/300‑1`  
         `aagACTTCATGTTGTCAATACCTGTTTTTCGTGCGACTTATCAGGCTGTCTACTTATCCGGTTGACCCCGGTGTCGATTGGGATGGCTATTCGCCGTGTCCCTCTCGATGGCTGTAAGTATCCTATAGGTTAGACTTTAAGTCAATACTCTTTTTGATAAATTTTAAATTAATCTTTTAAGTCTCTTTAAGTTGTCTCTCCTTA‑TAGTGAGTTGTATTAATAAAGACAATGATTAAAGGTTATCACTTTATGGCTAACAACAAGGCGTCTTTAGGTCTGGTCTTTATGTAG‑TGTCTTTA‑‑‑‑‑GG                                                                                                                                                                                                                                                                                                                    <  1:782975/298‑1`  
         `GTTACTTCATGTTGTCAATACCTGTTTTTCGTGCGACTTATCAGGCTGTTTACTTATCCGGTTGACCCCGGTGTCGATTGGGATGGCTATTCGCCGTGTCCCTCTCGATGGCTGTAAGTATCCTATAGTTTAGACTTTAAGTCAATACTCTTTTTGATAAATTTTAAATTAATCTTTTAAGTCTCTTTAAGTTGTCTCTCCTTA‑TAGTGAGTTGTATTAATAAAGACAATGATTAAAGGTTATCACTTTATGGCTAACAACAAGGCGTCTTTAGGTCTGGTCTTTATGTAG‑TGTCTTTA‑‑‑‑‑GG                                                                                                                                                                                                                                                                                                                    <  1:581316/301‑1`  
         `GTTACTTCATGTTGTCAATACCTGTTTTTCGTGCGACTTATCAGGCTGTCTACTTATCCGGTTGACCCCGGTGTCGATTGGGATGGCTATTCGCCGTGTCCCTCTCGATGGCTGTAAGTATCCTATAGGTTAGACTTTAAGTCAATACTCTTTTTGATAAATTTTAAATTAATCTTTTAAGTCTCTTTAAGTTGTCTCTCCTTA‑TAGTGAGTTGTATTAATAAAGACAATGATTAAAGGTTATCACTTTATGGCTAACAACAAGGCGTCTTTAGGTCTGGTCTTTATGTAG‑TGTCTTTA‑‑‑‑‑GG                                                                                                                                                                                                                                                                                                                    <  1:774939/301‑1`  
          `TTACTTCATGTTGTCAATACCTGTTTTTCGTGCGACTTATCAGGCTGTCTACTTATCCGGTTGACCCCGGTGTCGATTGGGATGGCTATTCGCCGTGTCCCTCTCGATGGCTGTAAGTATCCTATAGGTTAGACTTTAAGTCAATACTCTTTTTGATAAATTTTAAATTAATCTTTTAAGTCTCTTTAAGTTGTCTCTCCTTA‑TAGTGAGTTGTATTAATAAAGACAATGATTAAAGGTTATCACTTTATGGCTAACAACAAGGCGTCTTTAGGTCTGGTCTTTATTTAG‑TGTATTTA‑‑‑‑‑GGc                                                                                                                                                                                                                                                                                                                   >  1:255228/1‑300`  
          `TTACTTCATGTTGTCAATACCTGTTTTTCGTGCGACTTATCAGGCTGTCTACTTATCCGGTTGACCCCGGTGTCGATTGGGATGGCTATTCGCCGTGTCCCTCTCGATGGCTGTAAGTATCCTATAGGTTAGACTTTAAGTCAATACTCTTTTTGATAAATTTTAAATTAATCTTTTAAGTCTCTTTAAGTTGTCTCTCCTTA‑TAGTGAGTTGTATTAATAAAGACAATGATTAAAGGTTATCACTTTATGGCTAACAACAAGGCGTCTTTAGGTCTGGTCTTTATGTAG‑TGTCTTTA‑‑‑‑‑GGT                                                                                                                                                                                                                                                                                                                   <  1:707730/301‑1`  
          `TTACTTCATGTTGTCAATACCTGTTTTTCGTGCGACTTATCAGGCTGTCTACTTATCCGGTTGACCCCGGTGTCGATTGGGATGGCTATTCGCCGTGTCCCTCTCGATGGCTGTAAGTATCCTATAGGTTAGACTTTAAGTCAATACTCTTTTTGATAAATTTTAAATTAATCTTTTAAGTCTCTTTAAGTTGTCTCTCCTTA‑TAGTGAGTTGTATTAATAAAGACAATGATTAAAGGTTATCACTTTATGGCTAACAACAAGGCGTCTTTAGGTCTGGTCTTTATGTAG‑TGTCTTTA‑‑‑‑‑GGT                                                                                                                                                                                                                                                                                                                   <  1:564543/301‑1`  
           `gACTTCATGTTGTAAATACCTGTTTTTCGTGCGACTTATCAGGCTGTCTACTTATCCGGTTGACCCCGGTGTTGATTGGGATGGCTATTCGCCGTGTCCCTCTCGATGGCTGTAAGTATCCTATAGGTTAGACTTTAAGTCAATACTCTTTTTGATAAATTTTAAATTAATCTTTTAAGTCTCTTTAAGTTGTCTCTCCTTA‑TAGTGAGTTGTATTAATAAAGACAATGATTAAAGGTTATCACTTTATGGCTAACAACAAGGCGTCTTTAGGTCTGGTCTTTATGTAG‑TGTCTTTA‑‑‑‑‑GGTC                                                                                                                                                                                                                                                                                                                  <  1:467074/300‑1`  
           `ataagagacagGTCAATACCTGTTTTTCGTGCGACTTATCAGGCTGTCTACTTATCCGGTTGACCCCGGTGTCGATTGGGATGGCTATTCGCCGTGTCCCTCTCGATGGCTGTAAGTATCCTATAGGTTAGACTTTAAGTCAATACTATTTTTGATAAATTTTAAATTAATCTTTTAAGTCTCTTTAAGTTGTCTCTCCTTA‑TAGTGAGTTGTATTAATAAAGACAATGATTAAAGGTTATCACTTTATGGCTAACAACAAGGCGTCTTTAGGTCTGGTCTTTATGTAG‑TGTCTTTA‑‑‑‑‑GGTC                                                                                                                                                                                                                                                                                                                  <  1:248176/290‑1`  
           `TACTTCATGTTGTCAATACCTGTTTTTCGTGCGACTTATCAGGCTGTCTACTTATTCGGTTGACCCCGGTTTCGATTGGGATGGCTATTCGCCGTGTCCCTCTCGATGGCTGTAAGTATCCTATAGGTTAGACTTTAAGTCAATACTCTTTTTGATAAATTTTAAATTAATCTTTTAAGTCTCTTTAAGTTGTCTCTCCTTA‑TAGTGAGTTGTATTAATAAAGACAATGATTAAAGGTTATCACTTTATGGCTAACAACAAGGCGTCTTTAGGTCTGGTCTTTATGTAG‑TGTCTTTA‑‑‑‑‑GGTC                                                                                                                                                                                                                                                                                                                  <  1:532762/301‑1`  
           `TACTTCATGTTGTCAATACCTGTTTTTCGTGCGACTTATCAGGCTGTCTACTTATCCGGTTGACCCCGGTGTCGATTGGGATGGCTATTCGCCGTGTCCCTCTCGATGGCTGTAAGTATCCTATAGGTTAGACTTTAAGTCAATACTCTTTTTGATAACTTTTAAATTAATCTTTTAAGTCTCTTTAAGTTGTCTCTCCTTA‑TAGTGAGTTGTATTAATAAAGACAATGATTAAAGGTTATCACTTTATGGCTACCAACCAGGCGTCTTTAGGTCTGGTCTTTATGTCG‑TGTCTTTA‑‑‑‑‑GGTt                                                                                                                                                                                                                                                                                                                  >  1:222119/1‑300`  
           `TACTTCATGTTGTCAATACCTGTTTTTCGTGCGACTTATCAGGCTGTCTACTTATCCGGTTGACCCCGGTGTCGATTGGGATGGCTATTCGCCGTGTCCCTCTCGATGGCTGTAAGTATCCTATAGGTTAGACTTTAAGTCAATACTCTTTTTGATAAATTTTAAATTAATCTTTTAAGTCTCTTTAAGTTGTCTCTCCTTA‑TAGTGAGTTGTATTAATAAAGACAATGATTAAAGGTTATCACTTTATGGCTAACAACAAGGCGTCTTTAGGTCTGGTCTTTATGTAG‑TGTCTTTA‑‑‑‑‑GGTC                                                                                                                                                                                                                                                                                                                  >  1:691428/1‑301`  
           `TACTTCATGTTGTCAATACCTGTTTTTCGTGCGACTTATCAGGCTGTCTACTTATCCGGTTGACCCCGGTGTCGATTGGGATGGCTATTCGCCGTGTCCCTCTCGATGGCTGTAAGTATCCTATAGGTTAGACTTTAAGTCAATACTCTTTTTGATAAATTTTAAATTAATCTTTTAAGTCTCTTTAAGTTGTCTCTCCTTA‑TAGTGAGTTGTATTAATAAAGACAATGATTAAAGGTTATCACTTTATGGCTAACAACAAGGCGTCTTTAGGTATGGTCTTTATGTAG‑TGTCTTTA‑‑‑‑‑GGTt                                                                                                                                                                                                                                                                                                                  >  1:213738/1‑300`  
           `TACTTCATGTTGTCAATACCTGTTTTTCGTGCGACTTATCAGGCTGTCTACTTATCCGGTTGACCCCGGTGTCGATCGGTATGGCTATTCGCCGTGTCCCTCTCGATGGCTGTAAGTATCCTATAGGTTAGACTTTAAGTCAATACTCTTTTTGATAAATTTTAAATTAATCTTTTAAGTCTCTTTAAGTTGTCTCTCCTTA‑TAGTGAGTTGTATTAATAAAGACAATGATTAAAGGTTATCACTTTATGGCTAACAACAAGGCGTCTTTAGGTCTGGTCTTTATGTAG‑TGTCTTTA‑‑‑‑‑GGTt                                                                                                                                                                                                                                                                                                                  >  1:507036/1‑300`  
           `TACTTCATGTTGTCAATACCTGTTATTCGTGCGACTTATCAGGCTGTCTACTTATCCGGTTGACCCCGGTGTCGATTGGGATGGCTATTCGCCGTGTCCCTCTCGATGGCTGTAAGTATCCTAGAGGTTAGACTTTAAGTCAATACTCTTTTTGATAAATTTTAAATTAATCTTTTAAGTCTCTTTAAGTTGTCTCTCCTTA‑TAGTGAGTTGTATTAATAAAGACAATGATTAAAGGTTATCACTTTATGGCTAACAACAAGGCGTCTTTAGGTCTGGTCTTTATGTAG‑TGTCTTTA‑‑‑‑‑GGTt                                                                                                                                                                                                                                                                                                                  >  1:235100/1‑300`  
           `TACTTAATGTTGTCAATACCGGTTTTTCGTGCGACTTATCAGGCTGTCTACTTATCCGGTTGACCCCGGTGTCGATTAGGATGGCTATTCGCCGTGTCCCTCTCGATGGCTGTAAGTATCCTATAGGTTAGACTTTAAGTCAATACTCTTTTTGATAAATTTTAAATTAATCTTTTAAGTCTCTTTAAGTTGTCTCTCCTTA‑TAGTGAGTTGTATTAATAAAGACAATGATTAAAGGTTATCACTTTATGGCTAACAACAAGGCGTCTTTAGGTCTGGTCTTTATGTAG‑TGTCTTTA‑‑‑‑‑GGTC                                                                                                                                                                                                                                                                                                                  <  1:751185/301‑1`  
            `ACTTCATGTTGTCAATACCTGTTTTTCGTGCGACTTATCAGGCTGTCTACTTATCCGGTTGACCCCGGTGTCGATTGGGATGGCTATTCGCCGTGTCCCTCTCGATGGCTGTAAGTATCCTATAGGTTAGACTTTAAGTCAATACTCTTTTTGATAAATTTTAAATTAATCTTTTAAGTCTCTTTAAGTTGTCTCTCCTTA‑TAGTGAGTTGTATTAATAAAGACAATGATTAAAGGTTATCACTTTATGGCTAACAACAGGGCGTCTTTACGTCTGGTCTTTATGTAG‑TGTCTTTA‑‑‑‑‑GTTCT                                                                                                                                                                                                                                                                                                                 >  1:423195/1‑301`  
            `ACTTCATGTTGTCAATACCTGTTTTTCGTGCGACTTATCAGGCTGTCTACTTATCCGGTTGACCCCGGTGTCGATTGGGATGGCTATTCGCCGTGTCCCTCTCGATGGCTGTAAGTATCCTATAGGTTAGACTTTAAGTCAATACTCTTTTTGATAAATTTTAAATTAATCTTTTAAGTCTCTTTAAGTTGTCTCTCCTTA‑TAGTGAGTTGTATTAATAAAGACAATGATTAAAGGTTATCACTTTATGGCTAACAACAAGGCGTCTTTAGGTCTGGTCTTTATGTAG‑TGTCTTTA‑‑‑‑‑GGTCT                                                                                                                                                                                                                                                                                                                 >  1:115992/1‑301`  
            `ACTTCATGTTGTCAATACCTGTTTTTCGTGCGACTTATCAGGCTGTCTACTTATCCGGTTGACCCCGGTGTCGATTGGGATGGCTATTCGCCGTGTCCCTCTCAATGGCTGTAAGTATCCTATAGGTTAGACTTTAAGTCAATACTCTTTTTGATAAATTTTAAATTAATCTTTTAAGTCTCTTTAAGTTGTCTCTCCTTA‑TAGTGAGTTGTATTAATAAAGACAATGATTAAAGGTTATCACTTTATGGCTAACAACAAGGCGTCTTTAGGTCTGGTCTTTATGTAG‑TGTCTTTA‑‑‑‑‑GGTCT                                                                                                                                                                                                                                                                                                                 >  1:418818/1‑301`  
             `CTTCATGTTGTCAATACCTGTTTTTCGTGCGACTTATCAGGCTGTCTACTTATCCGGTTGACCCCGGTGTCGATTGGGATGGCTATTCGCCGTGTCCCTCTCGATGGCTGTAAGTATCCTATAGGTTAGACTTTAAGTCAATACTCTTTTTGATAAATTTTAAATTAATCTGTTAAGTCTCTTTAAGTTGTCTCTCCTTA‑TAGTGAGTTGTATTAATAAAGACAATGATTAAAGGTTATCACTTTATGGCTAACAACAAGGCGTCTTTAGGTCTGGTCTTTATGTAG‑TTTCTTTA‑‑‑‑‑GGTCTG                                                                                                                                                                                                                                                                                                                >  1:93746/1‑301`  
             `CTTCATGTTGTCAATACCTGTGTTTCGTGCGACTTATCAGGCTGTCTACTTATCCGGTTGACCCCGGTGTCGATTGGGATGGCTATTCGCCGTGTCCCTCTCGATGGCTGTAAGTATCCTATAGGTTAGACTTTAAGTCAATACTCTTTTTGATAAATTTTACATTAATCTTTTAAGTCTCTTTAAGTTGTCTCTCCTTA‑TAGTGAGTTGTATTAATAAAGACAATGATTAAAGGTTATCACTTTATGGCTAACAACAAGGCGTATTTAGGTCTGGTCTTTATGGAG‑TGTCTTTA‑‑‑‑‑GGTCTG                                                                                                                                                                                                                                                                                                                >  1:525507/1‑301`  
              `TTCATGTTGTCAATACCTGTTTTTCGTGCGACTTATCAGGCTGTCTACTTATCCGGTTGACCCCGGTGTCGATTGGGATGGCTATTCGCCGTGTCCCTCTCGATGGCTGTAAGTATCCTATAGGTTAGACTTTAAGTCAATACTCTTTTTGATAAATTTTAAATTAATCTTTTAAGTCTCTTTAAGTTGTCTCTCCTTA‑TAGTGAGTTGTATTAATAAAGACAATGATTAAAGGTTATCACTTTATGGCTAACAACAAGGCGTCTTTAGGTCTGGTCTTTATGTAG‑TGTCTTTA‑‑‑‑‑GGTCTGG                                                                                                                                                                                                                                                                                                               >  1:392085/1‑301`  
                `CATTTTGTCAATACCTGTTTTTCGTGCGACTTATCAGGCTGTCTACTTATCCGGTTGACCCCGGTGTCGATTGGGATGGCTATTCGCCGTGTCCCTCTCGATGGCTGTAAGTATCCTATAGGTTAGACTTTAAGTCAATACTCTTTTTGATAAATTTTAAATGAATCTTTTAAGTCTCTTTAAGTTGTCTCTCCTTA‑TAGTGAGTTGTATTAATAAAGACAATGATTAAAGGTTATCACTTTATGGCTAACAACAAGGCGTCTTTAGGTCTGGTCTTTATGTAG‑TGTCTTTA‑‑‑‑‑GGTCTGGTC                                                                                                                                                                                                                                                                                                             <  1:435573/301‑1`  
                `CATGTTGTCAATACCTGTTTTTCGTGCGACTTATCAGGCTGTCTACTTATCCGGTTGACCCCGGTGTCGATTGGGATGGCTATTCGCCGTGTCCCTCTCGATGGCTGTAAGTATCCTATAGGTTAGACTTTAAGTCAATACTCTTTTTGATAAATTTTAAATTAATCTTTTAAGTCTCTTTAAGTTGTCTCTCCTTA‑TAGTGAGTTGTATTAATAAAGACAATGATTAAAGGTTATCACTTTATGGCTAACAACAAGGCGTCTTTAGGTCTGGTCTTTATGTAG‑TGTCTTTA‑‑‑‑‑GGTCTGGTC                                                                                                                                                                                                                                                                                                             <  1:125517/301‑1`  
                `CATGTTGTCAATACCTGTTTTTCGTGCGACTTATCAGGCTGTCTACTTATCCGGTTGACCCCGGTGTCGATTGGGATGGCTATTCGCCGTGTCCCTATCGATGGCTGTAAGTATCCTATAGGTTAGACTTTAAGTCAATACTCTTTTTGATAAATTTTAAATTAATCTTTTAAGTCTCTTTAAGTTGTCTCTCCTTA‑TAGTGAGTTGTATTAATAAAGACAATGATTAAAGGTTATCACTTTATGGCTAACAACAAGGCGTCTTTAGGTCTGGTCTTTATGTAG‑TGTCTTTA‑‑‑‑‑GGTCTGGTC                                                                                                                                                                                                                                                                                                             <  1:395458/301‑1`  
                `CATGTTGTCAATACCTGTTTTTCGTGCGACTTATCAGGCTGTCTACTTATCCGGTTGACCCCGGTGTCGATTGGGATGGCTATTCACCGTGTCCCTCTCGATGGCTGTAAGTATCCTATAGGTTAGACTTTAAGTCAATACTCTTTTTGATAAATTTTAAATTAATCTTTTAAGTCTCTTTAAGTTGTCTCTCCTTA‑TAGTGAGTTGTATTAATAAAGACAATGATTAAAGGTTATCACTTTATGGCTAACAACAAGGCGTCTTTAGGTCTGGTCTTTATGTAG‑TGTCTTTA‑‑‑‑‑GGTCTGGTC                                                                                                                                                                                                                                                                                                             <  1:138925/301‑1`  
                `CATGTTGTCAATACCTGATTTTCGTGCGACTTATCAGGCTGTCTACTTATCCGGTTGACCCCGGTGTCGATTGGGATGGCTATTCGCCGTGTCCCTCTCGATGGCTGTAAGTATCCTATAGGTTAGACTTAAAGTCAATACTCTTTTTGATAAATTTTAAATTAATCTTTTAAGTCTCTTTAAGTTGTCTCTCCTTA‑TAGTGAGTTGTATTAATAAAGACAATGATTAAAGGTTATCACTTTATGGCTAACAACAAGGCGTCTTTAGGTCTGGTCTTTATGTAG‑TGTCTTTA‑‑‑‑‑GGTCTGGTt                                                                                                                                                                                                                                                                                                             >  1:616785/1‑300`  
                   `GTTGTCAATACCTGTTTTTCGTGCGACTTATCAGGCTGTCTACTTATCCGGTTGACCCCGGTGTCGATTGGGATGGCTATTCGCCGTGTCCCTCTCGATGGCTGTAAGTATCCTATAGGTTAGACTTTAAGTCAATACTCTTTTTGATAAATTTTAAATTAATCTTTTAAGTCTCTTTAAGTTGTCTCTCCTTA‑TAGTGAGTTGTATTAATAAAGACAATGATTAAAGGTTATCACTTTATGGCTAACAACAAGGCGTCTTTAGGTCTGGTCTTTATGTAG‑TGTCTTTA‑‑‑‑‑GGTCTGGTCTgt                                                                                                                                                                                                                                                                                                          >  1:174601/1‑299`  
                    `TTGTCAATACCTGCTTTTCGTGCGACTTATCAGGCTGTCTACTTATCCGGTTGACCCCGGTGTCGATTGGGATGGCTATTCGCCGTGTCCCTCTCGATGGCTGTACGTATCCTATAGGTTAGACTTTAAGTCAATACTCTTTTTGATTAATTTTAAATTCATCTTTTAAGTCTCTTTAAGTTGTCTCTCCTTA‑TAGTGAGTTGTATTAATAAAGACACTGATTAAAGGTTATCACTTTATGGCTAACCACAAGTCGTCTTTAGGTCTGGTCTTTATGTAC‑TCTCTTTA‑‑‑‑‑GGTCTGGTCTTTt                                                                                                                                                                                                                                                                                                         >  1:788897/1‑300`  
                      `GTCAATACCTGTTTTTCGTGCGACTTATCAGGCTGTCTACTTATCCGGTTGACCCCGGTGTCGATTGGGATGGCTATTCGCCGTGTCCCTCTCGATGGCTGTAAGTATCCTATAGGTTAGACTTTAAGTCAATACTCTTTTTGATAAATGTTAAATTAATCTTTTAAGTCTCTTTAAGTTGTCTCTCCTTA‑TAGTGAGTTGTATTAATAAAGACAATGATTAAAGGTTATCACTTTATGGCTAACAACAAGGCGTCTTTAGGTCTGGTCTTTATGTAG‑TGTCTTTA‑‑‑‑‑GGctgtctcttatac                                                                                                                                                                                                                                                                                                       >  1:187415/1‑288`  
                             `CCTGTTTTTCGTGCGACTTATCAGGCTGTCTACTTATCCGGTTGACCCCGGTGTCGATTGGGATGGCTATTCGCCGTGTCCCTCTCGATGGCTGTAAGTATCCTATAGGTTAGACTTTAAGTCAATACTCTTTTTGATAATTATTAAATTAATCTTTTAGGTCCTTTTAAGTT‑TTTCTCCCTATTGGTGAGTTGTTTTATTAAAGACAATGATTAAAGTTTATAACTTAATGGTTAACAACAAGGCGCCTTTAGGTCGGGTCTTTTTGTAG‑TGTCTTTG‑‑‑‑‑GGTCTGTTTTTTgggctggttt                                                                                                                                                                                                                                                                                                >  1:306877/1‑291`  
                             `CCTGTTTTTCGTGCGACTTATCAGGCTGTCTACTTATCCGGTTGACCCCGGTGTCGATTGGGATGGCTATTCGCCGTGTCCCTCTCGATGGCTGTAAGTATCCTATAGGTTAGACTTTAAGTCAATACTCTTTTTGATAAATTTTAAATTAATCTTTTAAGTCTCTTTAAGTTGTCTCTCCTTA‑TAGTGAGTTGTATTAATAAAGACAATGATTAAAGGTTATCACTTTATGGCTAACAACAAGGCGTCTTTAGGTCTGGTCTTTATGTAG‑TGTCTTTA‑‑‑‑‑GGctgtctcttatacacatctc                                                                                                                                                                                                                                                                                                >  1:629049/1‑281`  
                                     `TCGTGCGACTTATCAGGCTGTCTACTTATCCGGTTGACCCCGGTGTCGATTGGGATGGCTATTCGCCGTGTCCCTCTCGATGGCTGTAAGTATCCTATAGGTTAGACTTTAAGTCAATACTCTTTTTGATAAATTTTAAATTAATCTTTTAAGTCTCTTTAAGTTGTCTCTCCTTA‑TAGTGAGTTGTATTAATAAAGACAATGATTAAAGGTTATCACTTTATGGCTAACAACAAGGCGTCTTTAGGTCTGGTCTTTATGTAG‑TGTCTTTA‑‑‑‑‑GGctgtctcttatacacatctccgagccca                                                                                                                                                                                                                                                                                        >  1:89231/1‑273`  
                                                                    `GGTTGACCCCGGTGTCGATTGGGATGGCTATTCGCCGTGTCCCTCTCGATGGCTGTAAGTATCCTATAGGTTAGACTTTAAGTCAATACTCTTTTTGATAAATTTTAAATTAATCTTTTAAGTCTCTTTAAGTTGTCTCTCCTTA‑TAGTGAGTTGTATTAATAAAGACAATGATTAAAGGTTATCACTTTATGGCTAACAACAAGGCGTCTTTAGGTCTGGTCTTTATGTAG‑TGTCTTTAGGTCTGGTCTGGTCTTTATGTTTAAACTTTAAGATAGGCGTTGACTTGATGGGTCTTTAGG                                                                                                                                                                                                                                                              <  1:732562/301‑1`  
                                                                                                                                                                                         `TTAAGTCTCTTTAAGTTGTCTCTCCTTA‑TAGTGAGTTGTATTAATAAAGACAATGATTAAAGGTTATCACTTTATGGCTAACAACAAGGCGTCTTTAGGTCTGGTCTTTATGTAG‑TGTCTTTA‑‑‑‑‑GGTCTGGTCTTTATGTTTAAACTTTAAGATAGGCGTTGACTTGATGGGTCTTTAGGTGTAGGCTTTAGGTGTTGGCTTTAGGATGGACGTTAGGAGGTGACTTTAGGAGGATACTTTAGGAGACTGTAACAGATA‑GGGACACAGAGAGACACTCAAGGTAACACCCAAAGACAAACCC                                                                                                                                   <  1:453500/301‑1`  
                                                                                                                                                                                                                                                                                                 `tgtgtataagggacagCTTTA‑‑‑‑‑GGTCTGGTCTTTATGTTTAAACTTTAAGATAGGCGTTGACTTGATGGGTCTTTAGGTGTAGGCTTTAGGTGTTGGCTTTAGGATGGACGTTAGGAGGTTACTTTAGGAGTATACTTTAGGAGACTGTAACAGATA‑GGGACACAGAGAGACACTCAAGGTAACACCCAAAGACAAACCCAAGGGTCATCCCCAACCCTTAGGGAACCCTCAAGGTCAACCGAATGTTGACTTTAGGTGATTGGCTGGGCTTTAGGTACCCCCTATGGGGGAACTTTAGGTC                             <  1:150508/285‑1`  
                                                                                                                                                                                                                                                                                                 `tatgtataagagacagCTTTA‑‑‑‑‑GGTCTGGTCTTTATGTTTAAACTTTAAGATAGGCGTTGACTTGATGGGTCTTTAGGTGTAGGCTTTAGGTGTTGGCTTTAGGATGGACGTTAGGAGGTGACTTTAGGAGGATACTTTAGGAGACTGTAACAGATA‑GGGACACAGAGAGACACTCAAGGTAACACCCAAAGACAAACCCAAGGGTCATCCCCAACCCTTAGGGAACCCTCAAGGTCAACCGAAGGTTGACTTTAGGTGATTGGCTGGGCTTTAGGTACCCCCTATGGGGGAACTTTAGGTC                             <  1:804461/285‑1`  
                                                                                                                                                                                                                                                                                                      `taggtcG‑TGTCTTTA‑‑‑‑‑GGTCTGGTCGTTATGGTTAAACTTTAAGATAGGCGTTGACTTGAT‑GGTCTTTAGGTGTAGGCTTTAGGTGTTGGCTTTAGGATGGACGTTAGGAGGTGACTTTAGGAGGATACTGTAGGAGACTGTAACAGATA‑GGGACACAGAGAGACACTCAAGGTAACACCCAAAGACAAACCCAAGGATCATCCCCAACCCTTAGGGAACCCTCAAGGTCAACCGAAGGTTGACTTTAGGTGATTGGCTGGGCTTTAGGTACCCCCTATGGGGGAACTTTAGGTCCGTACAC                      <  1:242646/295‑1`  
                                                                                                                                                                                                                                                                                                      `gtgggctcggagatgggtataagagacagCTTTATGTTTAAACTTTAAGATAGGCGTTGAATTGATGGGTCTTTAGGTGTAGGCTTTAGGTGTTGGCTTTAGGATGGACGTTAGGAGGTGACTTTAGGAGGATACTTTAGGAGACTGTAACAGATA‑GGGACACAGAGAGACACTCAAGGTAACACCCAAAGACAAACCAAAGGGTCATCCCCAACCCTTAGGGAACCCTCAAGGTCAACCGAAGGTTGACTTTAGGTGATTGGCTGGGCTTTAGGTACCCCCTATGGGGGAACTTTAGGTC                             <  1:486625/272‑1`  
                                                                                                                                                                                                                                                                                                      `ggagatgggtataagagacagGGTCTGGTCTTTATGTTTAAACTTTAAGATAGGCGTTGAATTGATGGGTCTTTAGGTGTAGGCTTTAGGTGTTGGCTTTAGGATGGACGTTAGGAGGTGACTTTAGGAGGATACTTTAGGAGACTGTAACAGATC‑GGGACACAGAGAGACACTCAAGGTAACACCCAAAGACAAACCCAAGGGTCATCCCCAACCCTTAGGGAACCCTCAAGGTCAACCGAAGGTTGACTTTAGGTGATTGGCTGGGCTTTAGGTACCCCCTATGGGGGAACTTTAGGTC                             <  1:476554/280‑1`  
                                                                                                                                                                                                                                                                                                      `aagagacagtTCTTTA‑‑‑‑‑GGTCTGGTCTTTATGTTGAAACTTTAAGATAGGCGTTGACTTGATGGGTCTTTAGGTGTAGGCTTTAGGTGTTGGCTTTAGGATGGACGTTAGGAGGTGACTTTAGGAGGATACTTTAGGAGACTGTAACAGATC‑GGGACACAGAGAGACACTCAAGGTAACACCCAAAGACAAACCCAAGGTTCATCCCCAACCCTTAGGGAACCCTCAAGGTCAACCGAAGGTTGACTTTAGGTGATTGGCTGGGCTTTAGGTACCCCCTATGGGGGAACTTTAGGTCCGTAC                        <  1:442871/291‑1`  
                                                                                                                                                                                                                                                                                                       `taggtcttggtTTTA‑‑‑‑‑CGTCTGGTCTTTATGTTTAAACTTTAAGATCGGCGTTGACGTGATGGGTCTTTAGGTGTAGGCTTTAGGTGTTTGCTTTAGGATTGACGTTAGTAGGTGACTTTAGGAGGATACTTTAGGAGACTGCAACAGATA‑GTGCCACAGAGAGACACTCAAGGTAACACCCAAAGACAAACCCAAGCGTCATCCCCAACCCTTAGGGAACCCTCAAGGTCAACCGAAGGTTGACTTTAGGTGATTGGCTTGGCTTTAGGTA‑CCCCTATGGGGGAACTTTAGGTCCGTACAC                      <  1:69917/290‑1`  
                                                                                                                                                                                                                                                                                                        `gtgtataagagacA‑‑‑‑‑GGTCTGGTCTTTATGTTTAAACTTTAAGATAGGCGTTGACTTGATGGGTCTTTAGGTGTAGGCTTTAGGTGTTGGCTTTAGGATGGACGTTAGGAGGTGACTTTAGGAGGATACTTTAGGAGACTGTAACAGATC‑GGGACACAGAGAGACACTCAAGGTAACACCCAAAGACAAACCCAAGGGTCATCCCCAACCCTTATGGAACCCTCAAGGTCAACCGAAGGTTGACTTTAGGTGATTGGCTGGGCTTTAGGTACCCCCTATGGGGGAACTTTAGGTCCGTACAC                      <  1:460896/288‑1`  
                                                                                                                                                                                                                                                                                                        `agggttggttgTTA‑‑‑‑‑GGTCTAGGCTTGATGTTGAAACTTTAAGAGAGGCTTTGACTTGATGGGTCTTTAGGTGTAGGCTTTAGGAGTTGGCTTTAGGATGGACGTTAGTAGGTGACTTTAGGAGGATACTTTAGGATACTGTAACAGATA‑GGGACACAGAGAGACACTCAAGGTAACACCCAAAGACAAACCCAAGGGTAATCCCCAACCATTAGGGAACCCTCAAGGTCAACCGAAGGTTGACTTTAGGTGATTGGCTGGGCTTTAGGTACCCCCTATGGGGGAACTTTAGGTCCGTACAC                      <  1:558963/290‑1`  
                                                                                                                                                                                                                                                                                                        `aagagacagCTTTA‑‑‑‑‑GGTCTGGTCTTTATGTTTAAACTTTAAGATAGGCGTTGACTTGATGGGTCTTTAGGTGTAGGCTTTAGGTGTTGGCTTTAGGATGGACGTTAGGAGGTGACTTTAGGAGGATACTTTAGGAGACTGTAACAGATA‑GGGACACAGAGAGACACTCAAGGTAACACCCAAAGACAAACCCAAGGGTCATCCCCAACCCTTATGGAACCCTCAAGGTCAACCGAAGGTTGACTTTAGGTGATTGGCTGGGCTTTAGGTACCCCCTATGGGGGAACTTTAGGTCCGTACAC                      <  1:410318/292‑1`  
                                                                                                                                                                                                                                                                                                        `aagagacagCTTTA‑‑‑‑‑GGTCTGGTCTTTATGTTTAAACTTTAAGATAGGCGTTGACTTGATGGGTCTTTAGGTGTAGGCTTTAGGTGTTGGCTTTAGGATGGACGTTAGGAGGTGACTTTAGGAGGATACTTTAGGAGACTGTAACAGATA‑GGGACACAGAGAGACACTCAAGGTAACACCCAAAGACAAACCCAAGGGTCATCCCCAACCCTTAGGGAACCCTCAAGGTCAACCGAAGGTTGACTTTAGGTGATTGGCTGGGCTTTAGGTACCCCCTATGGGGGAACTTTAGGTCCGTACAC                      <  1:265212/292‑1`  
                                                                                                                                                                                                                                                                                                        `aagagacagCTTTA‑‑‑‑‑GGTCTGGTCTTTATGTTTAAACTTTAAGATAGGCGTTGACTTGATGGGTCTTTAGGTGTAGGCTTTAGGTGTTGGCTTTAGGATGGACGTTAGGAGGTGACTTTAGGAGGATACTTTAGGAGACTGTAACAGATA‑GGGACACAGAGAGACACTCAAGGTAACACCCAAAGACAAACCCAAGGGTCATCCCCAACCCTTAGGGAACCCTCAAGGTCAACCGAAGGTTGACTTTAGGTGATTGGCTGGGCTTTAGGTACCCCCTATGGGGGAACTTTAGGTCCGTACAC                      <  1:661653/292‑1`  
                                                                                                                                                                                                                                                                                                          `cggagatgtgtataagagacagGGTCTTTATGTTTAAACTTTAAGATAGGCGTTGACTTGATGGGTCTTTAGGTGTAGGCTTTAGGTGTTGGCTTTAGGATGGACGTTAGGAGGTGACTTTAGGAGGATACTTTAGGAGACTGTAACAGATA‑‑GGACACAGAGAGACACTCAAGGTAACACCCAAAGACAAACCCAAGGGTCATCCCCAACCCTTAGGGAACCCTCAAGGTCAACCGAAGGTTGACTTTAGGTGATTGGCTGGGCTTTAGGTACCCCCTATGGGGGAACTTTAGGTCCGTAC                        <  1:591081/279‑1`  
                                                                                                                                                                                                                                                                                                          `atgtgtataagagacaggggCTGGTCTTTAATGTTAAACTTTAAGATAGGCGTTGACTTGATGGGTCTTTAGGTGTAGGCTTTAGGTGTTGGCTTTAGGATGGACGTTAGGAGGTGACTTTAGGAGGATATTTTAGGAGACTGGAACAGAT‑‑GGGACACAGAGAGACACTCAAGGTAACACCCAAAGACAAACCCAAGGGTCATCCCCAACCCTTAGGGAACCCTCAAGATCAACCGAAGGTTGACTTTAGGTGATTGGCTGGGCTTTAGGTACCCCATATGGGGGAACTTTAGGTCCGTAC                        <  1:369847/281‑1`  
                                                                                                                                                                                                                                                                                                           `tgtgtagaagagacagGGTATGGTCGTTATGTTTAAACTTTAAGATAGGCGTTGACTGGATGGGTCTTTAGGTGTAGGCTTTAGGTGTTGGCTTTAGGATGGACGTTAGGAGGTGACGTTAGGAGGATACTTTAGGAGACTGTAACAGATA‑GGGACACAGAGAGACACTCAAGGTAACACCCAAAGACAAACCCAAGGGTCATCCCCAACCCTTAGGGAACCCTCAAGGTCAACCGAAGGTTGACTTTAGGTGATTGGCTGGGCTTTAGGTACCCCCTATGGGGGAACTTTAGGTCCGTAC                        <  1:272246/285‑1`  
                                                                                                                                                                                                                                                                                                             `tgtataagggacagGGTCTGGTCTTTATGTTTAAACTTTAAGATAGGCGTTGACTTGATGGGTCTTTAGGTGTAGGCTTTAGGTGTTGGCTTTAGGATGGACGTTAGGAGGTGACTTTAGGAGGATACTTTAGGAGACTGGAACAGATA‑GGGACACAGAGAGACACTCAAGGTAACACCCAAAGACAAACCCAAGGGTCATCCCCAACCCTTAGGGAACCCTTAAGGTCAACCGAAGGTTGACTTTAGGTGATTGGCTGGGCTTTAGGTACCCCCTATGGGGGAACTTTAGGTCCGTACAC                      <  1:280128/287‑1`  
                                                                                                                                                                                                                                                                                                             `tgtataagagacaggttctgtgCTTTATGTTTAAACTTTAAGATAGGCGTTGACTTGATGGGTCTTTAGGTGTAGGCTTTATGTGTTGTCTTTAGGATGGACGTTAGGAGGTGACTTTAGGAGGATACTTTAGGAGACTGTAACAGATA‑GGGACACAGAGAGATACTCAATGTAACACCCAAAGACAAACCCAAGGGTCATCCCCAACCCTTAGGGAACCCTCAAGGTCAACCGAAGGTTGACTTTAGGTGATTGGCTGGGCTTTAGGTACCCCCTATGGGGGAACTTTAGGTCCGTACAC                      <  1:347969/279‑1`  
                                                                                                                                                                                                                                                                                                             `tgtataagagacagGGTCTGGTCTTTATGTTTAAACTTTAAGATAGGCGTTGACTTGATGGGTCTTTAGGTGTAGGCTTTAGGTGTTGGCTTTAGGATGGACGTTAGGAGGTGACTTTAGGAGGATACTTTAGGAGACTGTAACAGATA‑GGGACACAGAGAGACACTCAAGGTAACACCCAAAGACAAACCCAAGGGTCATCCCCAACCCTTAGGGAACCCTCAAGGTCAACCGAAGGTTGACTTTAGGTGATTGGCTGGGCTTTAGGTACCCCCTATGGGGGAACTTTAGGTCCGTACAC                      <  1:36423/287‑1`  
                                                                                                                                                                                                                                                                                                             `ggtataagagacagGGTCTGGTCTTTAGGTTTAAACTTTAAGATAGGCGTTGACTTTATGGGTCTTTAGGTGTAGGCTTTAGGTGTTGGCTTTAGGATGGACGTTAGGAGGTGACTTTAGGAGGATACTTTAGGAGACTGTAACAGATA‑GGGACACAGAGAGACACTCAAGGTAACACCCAAAGACAAACCCAAGGGTCATCCCCAACCCTTAGGGAACCCTCAAGGTCAACCGAAGGTTGACTTTAGGTGATTGGCTGGGCTTTATGTACCCCCTATGGGGGAACTTTAGGTCCGTACAC                      <  1:801303/287‑1`  
                                                                                                                                                                                                                                                                                                             `ggctcggagatgtgtataagagacagATGTTTAAACTTTAAGATAGGCGTTGACTTGATGGGTCTTTAGGTGTAGGCTTTAGGTGTTGGCTTTAGGATGTACGTTAGGAGGTGACTTTAGGAGGATACTTTAGGAGACTGTAACAGATA‑GGGACACAGAGAGACACTCAAGGTAACACCCAAAGACAAACCCAAGGGTCATCCCCAACCCTTAGGGAACCCTCAAGGTCAACCGAAGGTTGACTTTAGGTGATTGGCTGGGCTTTAGGTACCCCCTATGGGGGAACTTTAGGTCCGTACAC                      <  1:684730/275‑1`  
                                                                                                                                                                                                                                                                                                             `gagatgtgtataagagacaGGTCTTTATGTTTAAACTTTAAGATAGGCGTTGACTTGATGGGTCTTTAGGTGTAGGCTTTAGGTGTTGGCTTTAGGATGGACGTTAGGAGGTGACTTTAGGAGGATACTTTAGGAGACTGTAACAGATC‑GGGACACAGAGAGACACTCAAGGTAACACCCAAAGACAAACCCAAGGGTCATCCCCAACCCTTAGGGAACCCTCAAGGTCAACCGAAGGTTGACTTTAGGTGATTGGCAGGGCTTTAGGTACCCCCTATGGGGGAACTTTAGGTCCGTACAC                      <  1:426457/282‑1`  
                                                                                                                                                                                                                                                                                                             `gagatgtgtataagagacaGGTCTTTATGTTTAAACTTTAAGATAGGCGTTGACTTGATGGGTCTTTAGGTGTAGGCTTTAGGTGTTGGCTTTAGGATGGACGTTAGGAGGTGACTTTAGGAGGATACTTTAGGAGACTGTAACAGATA‑GGGACACAGAGAGACACTCAAGGTAACACCCAAAGACAAACCCAAGGGTCATCCCCAACCCTTAGGGAACCCTCAAGGTCAACCGAAGGTTGACTTTAGGTGATTGGCTGGGCTTTAGGTACCCCCTATGGGGGAACTTTAGGTCCGTACAC                      <  1:815476/282‑1`  
                                                                                                                                                                                                                                                                                                             `gagatgtgtataagagacaGGTCTTTATGTTTAAACTTTAAGATAGGCGTTGACGTGATGGGTCTTTAGGTGTAGGCTTTAGGTGTTGGCTTTAGGATGGACGTTAGGAGGTGACTTTAGGAGGATACTTTAGGAGACTGTAACAGATT‑GGGACACAGAGAGACACTCAAGGTAACACCCAAAGACAAACCCAAGGGTCATCCCCAACCCTTAGGGAACCCTCAAGGTCAACCGAAGGTTGACTTTAGGTGATTGGCTGGGCTTTAGGTACCCCCTATGGGGGAACTTTAGGTCCGTACAC                      <  1:385157/282‑1`  
                                                                                                                                                                                                                                                                                                             `agatgtgtataagagacagGGTCTTTATGTTTAAACTTTAAGATAGGCGTTGACTTGATGGGTCTTTAGGTGTAGGCTTTAGGTGTTGGCTTTAGGATGGACGTTAGGAGGTGACTTTAGGAGGATACTTTAGGAGACTGTAACAGATA‑GGGACACAGAGAGACACTCAAGGTAACACCCAAAGACAAACCCAAGGGTCATCCCCAACCCTTAGGGAACCCTCAAGGTCAACCGAAGGTTGACTTTAGGTGATTGGCTGGGCTTTAGGTACCCCCTATGGGGGAACTTTAGGTCCGTACAC                      <  1:650695/282‑1`  
                                                                                                                                                                                                                                                                                                               `GTCTTTA‑‑‑‑‑GGTCTGGTCTTTATGTTTAAACTTTAAGATAGGCGTTGACTTGATGGGTCTTTAGGTGTAGGCTTTAGGTGTTGGCTTTAGGATGGACGTTAGGAGGTGACTTTAGGAGGATACTTTAGGAGACTGTAACAGATC‑GGGACACAGAGAGACACTCAAGGTAACACCCAAAGACAAACCCAAGGGTCATCCCCAACCCTTAGGGAACCCTCAAGGTCAACCGAAGGTTGACTTTAGGTGATTGGCTGGGCTTTAGGTACCCCCTATGGGGGAACTTTAGGTCCGTACACctgtctc               >  1:320753/1‑294`  
                                                                                                                                                                                                                                                                                                               `GTCTTTA‑‑‑‑‑GGTCTGGTCTTTATGTTTAAACTTTAAGATAGGCGTTGACTTGATGGGTCTTTAGGTGTAGGCTTTAGGTGTTGGCTTTAGGATGGACGTTAGGAGGTGACTTTAGGAGGATACTTTAGGAGACTGTAACAGATC‑GGGACACAGAGAGACACTCAAGGTAACACCCAAAGACAAACCCAAGGGTCATCCCCAACCCTTAGGGAACCCTCAAGGTCAACCGAAGGTTGACTTTAGGTGATTGGCTGGGCTTTAGGTACCCCCTATGGGGGAACTTTAGGTCCGTACACctgtctc               >  1:83835/1‑294`  
                                                                                                                                                                                                                                                                                                                `TCTTTA‑‑‑‑‑GGTCTGGTCTTTATGTTTAAACTTTAAGAGAGGCGTTGACTTGATGGGTCTTTAGGTGTAGGCGTGAGGTGTTGGCTTTAGGATGGACGTTAGGAGGTGACTTTAGGAGGATACTTTAGGAGACTGTAACAGATA‑TGGACACAGAGAGACACTCAAGGTAACAACCAAAGACAAACCCAAGGGTCATCCCCAACCCTTAGGGAACCCTCAAGGTCAACCGAAGGTTGACTTTAGGTGATTGGCGGGGCTTTAGGTACCCCCTATGGGGGAACTTTAGGTCCGTACACTGTGAGAt              <  1:714766/301‑2`  
                                                                                                                                                                                                                                                                                                                 `CTTTA‑‑‑‑‑GGTCTGGTCTTTATGTTTAAACTTTAAGATAGGCGTTGACTTGATGGGTCTTTAGGTGTAGGCTTTAGGTGTTGGCTTTAGGATGGACGTTAGGAGGTGACTTTAGGAGGATACTTTAGGAGACTGTAACAGATC‑GGGACACAGAGAGACACTCAAGGTAACACCCAAAGACAAACCCAAGGGTCATCCCCAACCCTTAGGGAACCCTCAAGGTCAACCGAAGGTTGACTTTAGGTGATTGGCTGGGCTTTAGGTACCCCCTATGGGGGAACTTTAGGTCCtgtctcttatacaca             >  1:378960/1‑286`  
                                                                                                                                                                                                                                                                                                                 `CTTTA‑‑‑‑‑GGTCTGGTCTTTATGTTTAAACTTTAAGATAGGCGTTGACTTGATGGGTCTTTAGGTGTAGGCTTTAGGTGTTGGCTTTAGGATGGACGTTAGGAGGTGACTTTAGGAGGATACTTTAGGAGACTGTAACAGATC‑GGGACACAGAGAGACACTCAAGGTAACACCCAAAGACAAACCCAAGGGTCATCCCCAACCCTTAGGGAACCCTCAAGGTCAACCGAAGGTTGACTTTAGGTGATTGGCTGGGCTTTAGGTACCCCCTATGGGGGAACTTTAGGTCCGTACACctgtctctt             >  1:301801/1‑292`  
                                                                                                                                                                                                                                                                                                                 `CTTTA‑‑‑‑‑GGTCTGGTCTTTATGTTTAAACTTTAAGATAGGCGTTGACTTGATGGGTCTTTAGGTGTAGGCTTTAGGTGTTGGCTTTAGGATGGACGTTAGGAGGTGACTTTAGGAGGATACTTTAGGAGACTGTAACAGATA‑GGGACACAGAGAGACACTCAAGGTAACACCCAAAGACAAACCCAAGGGTCATCCCCAACCCTTAGGGAACCCTCAAGGTCAACCGAAGGTTGACTTTAGGTGATTGGCTGGGCTTTAGGTACCCCCTATGGGGGAACTTTAGGTCCGTACctgtctcttat             >  1:52062/1‑290`  
                                                                                                                                                                                                                                                                                                                 `CTTTA‑‑‑‑‑GGTCTGGTCTTTATGTTTAAACTTTAAGATAGGCGTTGACTTGATGGGTCTTTAGGTGTAGGCTTTAGGTGTTGGCTTTAGGATGGACGTTAGGAGGTGACTTTAGGAGGATACTTTAGGAGACTGTAACAGATA‑GGGACACAGAGAGACACTCAAGGTAACACCCAAAGACAAACCCAAGGGTCATCCCCAACCCTTAGGGAACCCTCAAGGTCAACCGAAGGTTGACTTTAGGTGATTGGCTGGGCTTTAGGTACCCCCTATGGGGGAACTTTAGGTCCGTACACctgtctctt             >  1:33833/1‑292`  
                                                                                                                                                                                                                                                                                                                 `CTTTA‑‑‑‑‑GGTCTGGTCTTTATGTTTAAACTTTAAGATAGGCGTTGACTTGATGGGTCTTTAGGTGTAGGCTTTAGGTGTTGGCTTTAGGATGGACGTTAGGAGGTGACTTTAGGAGGATAC‑TTAGGAGACTGTAACAGATA‑GGGACACAGAGAGACACTCAAGGTAACACCCAAAGACAAACCCAAGGGTCATCCCCAACCCTTAGGGAACCCTCAAGGTCAACCGAAGGTTGACTTTAGGTGACTGGCTGGGCTTTAGGTACCCCCTATGGGGGAACTTTAGGTCCGTACctgtctcttata            >  1:15636/1‑289`  
                                                                                                                                                                                                                                                                                                                           `GGTCTGGTCTTTATGTTTAAACTTTAAGATAGGCGTTGACTTGATGGGTCTTTAGGTGTAGGCTTTAGGTGTTGGCTTTAGGATGGACGTTAGGAGGTGACTTTAGGAGGATACTTTAGGAGACTGTAACAGATC‑GGGACACAGAGAGACACTCAAGGTAATACCCAAAGACAAACCCAAGGGTCATCCCCAACCCTTAGGGAACCCTCAAGGTCAACCGAAGGTTGACTTTAGGTGATTGGCTGGGCTTTAGGTACCCCCTATGGGGGAACTTTAGGTCCGTACACctgtctcttataca        >  1:600517/1‑287`  
                                                                                                                                                                                                                                                                                                                           `GGTCTGGTCTTTATGTTTAAACTTTAAGATAGGCGTTGACTTGATGGGTCTTTAGGTGTAGGCTTTAGGTGTTGGCTTTAGGATGGACGTTAGGAGGTGACTTTAGGAGGATACTTTAGGAGACTGTAACAGATA‑GGGACACAGAGAGACACTCAAGGTAACACCCAAAGACAAACACAAGGGTCATCCCCAACCCTTAGGGAACCCTCAAGGTCAACCGAAGGTTGACTTTAGGTGATTGGCTGGGCTTTAGGTACCCCCTATGGGGGAACTTTAGGTCCtgtctcttatacacatctcc        >  1:622010/1‑281`  
                                                                                                                                                                                                                                                                                                                           `GGTCTGGTCTTTATGTTTAAACTTTAAGATAGGAGTTGACTTGATGGGTCTTTAGGTGTAGGCTTTAGGTGTTGGCTTTAGGATGGACGTTAGGAGGTGACTTTAGGAGGATACTTTAGGAGACTGTAACAGATA‑GGGACACAGAGAGACACTCAAGGTAACACCCAAAGACAAACCCAAGGGTCATCCCCAACCCTTAGGGAACCCTCAAGGTCAACCGAAGGTTGACTTTAGGTGATTGGCTGGGCTTTAGGTACCCCCTATGGGGGAACTTTAGGTCCGTACACctgtctcttataca        >  1:167699/1‑287`  
                                                                                                                                                                                                                                                                                                                                `GGTCTTTATGTTTAAACTTTAAGATAGGCGTTGACTTGATGGGTCTTTAGGTGTAGGCTTTAGGTGTTGGCTTTAGGATGGACGTTAGGAGGTGACTTTAGGAGGATACTTTAGGAGACTGTAACAGATAGGGGACACAGAGAGACACTCAAGGTAACACCCAAAGACAAACCCAAGGGTCATCCCCAACCCTTAGGGAACCCTCAAGGTCAACCGAAGGTTGACTTTAGGTGATTGGCTGGGCTTTAGGTACCCCCTATGGGGGAACTTTAGGTCCtgtctcttatacacatctccgagc    >  1:658613/1‑277`  
                                                                                                                                                                                                                                                                                                                                `GGTCTTTATGTTTAAACTTTAAGATAGGCGTTGACTTGATGGGTCTTTAGGTGTAGGCTTTAGGTGTTGGCTTTAGGATGGACGTTAGGAGGTGACTTTAGGAGGATACTTTAGGAGACTGTAACAGATA‑GGGACACAGAGAGACACTCAAGGTAACACCCAAAGACAAACCCAAGGGTCATCCCCAACCCTTAGGGAACCCTCAAGGTCAACCGAAGGTTGACTTTAGGTGATTGGCTGGGCTTTAGGTACCCCCTATGGGGGAACTTTAGGTCCGTACACctgtctcttatacacatct   >  1:633182/1‑282`  
                                                                                                                                                                                                                                                                                                                                `GGTCTTTATGTTTAAACTTTAAGATAGGCGTTGACTTGATGGGTCTTTAGGTGTAGGCTTTAGGTGTTGGCTTTAGGATGGACGTTAGGAGGTGACTTTAGGAGGATACTTTAGGAGACTGGAACAGATA‑GGGACACAGAGAGACACTCAAGGTAACACCCAAAGACAAACCCAAGGGTCATCCCCAACCCTTAGGGAACCCTCAAGGTCAACCGAAGGTTGACTTTAGGTGATTGGCTGGGCTTTAGGTACCCCCTATGGGGGAACTTTAGGTCCGTACACctgtctcttatacacatct   >  1:179156/1‑282`  
                                                                                                                                                                                                                                                                                                                                `GGTCTTTATGTTTAAACTTTAAGATAGGCGTTGACTTGATGGGTCTTTAGGTGTAGGCTTTAGGTGTTGGCTTTAGGATGGACGTTAGGAGGTGAATTTAGGAGGATACTTTAGGAGACTGTAACAGATA‑GGGACACAGAGAGACACTCAAGGTAACACCCAAAGACAAAACCAAGGGTCATCCCCAACCCTTAGGGAACCCTCAAGGGCAACCGAAGGTTGACTTTAGGTGATTGGATGGGCTTTAGGTACCCCCTATGGGGGAACATTAGGTCCGTACACcggtctcttatacacattt   >  1:478790/1‑282`  
                                                                                                                                                                                                                                                                                                                                `GGTCTTTACGTTTAAACTTTAAGATAGGCGTTGACTTGATGGGTCTTTAGGTGTAGGCTTTAGGTGTTGGCTTTAGGATGGACGTTAGGAGGTGACTTTAGGAGGATACTTTAGGAGACTGTAACAGATA‑GGGACACAGAGAGACACTCAAGGTAACACCCAAAGACAAACCCAAGGGTCATCCCCAACCCTTAGGGAACCCTCAAGGTCAACCGAAGGTTGACTTTAGGTGATTGGCTGGGCTTTAGGTACCCCCTATGGGGGAACTTTAGGTCCtgtctcttatacacatctccgagcc   >  1:737189/1‑276`  
                                                                                                                                                                                                                                                                                                                                 `GTCTTTATGTTTAAACTTTAAGATAGGCGTTGACTTGATGGGTCTTTAGGTGTAGGCTTTAGGTGTTGGCTTTAGGATGGACGTTAGGAGGTGACTTTAGGAGGATACTTTAGGAGACTGTAACAGATA‑GGGACACAGAGAGACACTCAAGGTAACACCCAAAGACAAACCCAAGGGTCATCCCCAACCCTTAGGGAACCCTCAAGGTCAACCGAAGGTTGACTTTAGGTGATTGGCTGGGCTTTAGGTACCCCCTATGGGGGAACTTTAGGTCCGTACACctgtctcttatacacatctc  >  1:204559/1‑281`  
                                                                                                                                                                                                                                                                                                                                 `GTCTTTATGTTTAAACTTTAAGATAGGCGTTGACTTGATGGGTCTTTAGGTGTAGGCTTTAGGTGTTGGCTTTAGGATGGACGTTAGGAGGTGACTTTAGGAGGATACTTTAGGAGACTGTAACAGATA‑GGGACACAAAGAGACACTCAAGGTAACACCCAAAGACAAACCCAAGGGTCATCCCCAACCCTTAGGGAACCCTCAAGGTCAACCGAAGGTTGACTTTAGGTGATTGGCTGGGCTTTAGGTACCCCCTATGGGGGAACTTTAGGTCCtgtctcttatacacatctccgagccc  >  1:327395/1‑275`  
                                                                                                                                                                                                                                                                                                                                 `GTCTTTATGTTTAAACTTTAAGATAGGCGTTGACTTGATGGGTCTTTAGGTGTAGGCTTTAGGTGTTGGCTTTAGGATGGACGTTAGGAGGTGACTTTAGGAGGATACTTTAGGAGACTGGAACAGATA‑GGGACACAGAGAGACACTCAAGGTAACACCCAAAGACAAACCCAAGGGTCATCCCCAACCCTTAGGGAACCCTCAAGGTCAACCGAAGGTTGACTTTAGGTGATTGGCTGGGCTTTAGGTACCCCCTATGGGGGAACTTTAGGTCCGTACctgtctcttatacacatctccg  >  1:770096/1‑279`  
                                                                                                                                                                                                                                                                                                                                 `GTCTTTATGTTTAAACTTTAAGATAGGCGTTGACTTGATGGGTCTTTAGGTGTAGGCTTTAGGTGTTGGCTTTAGGATGGACGTTAGGAGGTGACTTTAGGAGGATACTTTAGGAGACTGGAACAGATA‑GGGACACAGAGAGACACTCAAGGTAACACCCAAAGACAAACCCAAGGGTCATCCCCAACCCTTAGGGAACCCTCAAGGTCAACCGAAGGTTGACTTTAGGTGATTGGCTGGGCTTTAGGTACCCCCTATGGGGGAACTTTAGGTCCGTACACctgtctcttatacacatatc  >  1:500113/1‑281`  

                                                                                                                                                                                                                                                                                                                                                                                                                                                                                                                                                                                                                                                 
`TTACTGCATGTTACTTCATGTTGTCAATACCTGTTTTTCGTGCGACTTATCAGGCTGTCTACTTATCCGGTTGACCCCGGTGTCGATTGGGATGGCTATTCGCCGTGTCCCTCTCGATGGCTGTAAGTATCCTATAGGTTAGACTTTAAGTCAATACTCTTTTTGATAAATTTTAAATTAATCTTTTAAGTCTCTTTAAGTTGTCTCTCCTTA‑TAGTGAGTTGTATTAATAAAGACAATGATTAAAGGTTATCACTTTATGGCTAACAACAAGGCGTCTTTAGGTCTGGTCTTTATGTAG‑TGTCTTTA‑‑‑‑‑GGTCTGGTCTTTA‑‑‑‑‑‑‑‑‑‑‑‑‑‑‑‑‑‑‑‑‑‑‑‑‑‑‑‑‑‑‑‑‑‑‑‑‑‑‑‑‑‑‑‑‑‑‑‑‑‑‑‑‑‑‑‑‑‑‑‑‑‑‑‑‑‑‑‑‑‑‑‑‑‑‑‑‑‑‑‑‑‑‑‑‑‑‑‑‑‑‑‑‑‑‑‑‑‑‑‑‑‑‑‑‑‑‑‑‑‑‑‑‑‑‑‑‑‑‑‑‑‑‑‑‑‑‑‑‑‑‑‑‑‑‑‑‑‑‑‑‑‑‑‑‑‑‑‑‑‑‑‑‑‑‑‑‑‑‑‑‑‑‑‑‑‑‑‑‑‑‑‑‑‑‑‑‑‑‑‑‑‑‑‑‑‑‑‑‑‑‑‑‑‑‑‑‑‑‑‑‑‑‑‑‑‑‑‑‑‑‑‑‑‑‑‑‑‑‑‑‑‑‑‑‑‑‑‑‑‑‑‑‑‑‑‑‑‑‑‑‑‑‑‑‑‑‑‑‑‑‑‑‑‑‑‑‑‑‑‑‑‑‑‑‑‑‑‑‑‑‑‑‑‑‑‑‑‑‑‑‑‑‑‑‑‑‑‑‑‑‑‑‑‑‑  <  NC_001604.1/615‑295`  
`‑‑‑‑‑‑‑‑‑‑‑‑‑‑‑‑‑‑‑‑‑‑‑‑‑‑‑‑‑‑‑‑‑‑‑‑‑‑‑‑‑‑‑‑‑‑‑‑‑‑‑‑‑‑‑‑‑‑‑‑‑‑‑‑‑‑‑‑‑‑‑‑‑‑‑‑‑‑‑‑‑‑‑‑‑‑‑‑‑‑‑‑‑‑‑‑‑‑‑‑‑‑‑‑‑‑‑‑‑‑‑‑‑‑‑‑‑‑‑‑‑‑‑‑‑‑‑‑‑‑‑‑‑‑‑‑‑‑‑‑‑‑‑‑‑‑‑‑‑‑‑‑‑‑‑‑‑‑‑‑‑‑‑‑‑‑‑‑‑‑‑‑‑‑‑‑‑‑‑‑‑‑‑‑‑‑‑‑‑‑‑‑‑‑‑‑‑‑‑‑‑‑‑‑‑‑‑‑‑‑‑‑‑‑‑‑‑‑‑‑‑‑‑‑‑‑‑‑‑‑‑‑‑‑‑‑‑‑‑‑‑‑‑‑‑‑‑‑‑‑‑‑‑‑‑‑‑‑‑‑‑‑‑‑‑‑‑‑‑‑‑‑‑‑‑‑‑‑‑‑‑‑‑‑‑‑‑‑‑‑‑‑‑‑‑‑‑‑‑‑‑‑‑gtcttta‑‑‑‑‑ggtctggtctttaTGTTTAAACTTTAAGATAGGCGTTGACTTGATGGGTCTTTAGGTGTAGGCTTTAGGTGTTGGCTTTAGGATGGACGTTAGGAGGTGACTTTAGGAGGATACTTTAGGAGACTGTAACAGATA‑GGGACACAGAGAGACACTCAAGGTAACACCCAAAGACAAACCCAAGGGTCATCCCCAACCCTTAGGGAACCCTCAAGGTCAACCGAAGGTTGACTTTAGGTGATTGGCTGGGCTTTAGGTACCCCCTATGGGGGAACTTTAGGTCCGTACACTGTGAGA‑‑‑‑‑‑‑‑‑‑‑‑‑  <  NC_001604.1/281‑1`

  

Alignment Legend

Aligned base mismatch/match (shaded by quality score): `ATCG/ATCG` `< 3 ≤` `ATCG/ATCG` `< 11 ≤` `ATCG/ATCG` `< 26 ≤` `ATCG/ATCG` `< 37 ≤` `ATCG/ATCG` `< 38 ≤` `ATCG/ATCG`

Unaligned base: `atcg`    Masked matching base: `atcg`    Alignment gap: `‑`    Deleted base: `‑`

Reads not counted as support for junction

read\_name Not counted due to insufficient overlap past the breakpoint.

read\_name Not counted due to not crossing MOB target site duplication.
